# An Integrated Analysis of Clinical, Genomic, and Imaging Features Reveals Predictors of Neurocognitive Outcomes in a Longitudinal Cohort of Pediatric Cancer Survivors, Enriched with CNS Tumors (Rad ART Pro)

**DOI:** 10.3389/fonc.2022.874317

**Published:** 2022-06-23

**Authors:** Cassie Kline, Schuyler Stoller, Lennox Byer, David Samuel, Janine M. Lupo, Melanie A. Morrison, Andreas M. Rauschecker, Pierre Nedelec, Walter Faig, Dena B. Dubal, Heather J. Fullerton, Sabine Mueller

**Affiliations:** ^1^ Division of Oncology, Department of Pediatrics, Children’s Hospital of Philadelphia, University of Pennsylvania Perelman School of Medicine, Philadelphia, PA, United States; ^2^ Division of Child Neurology, Department of Neurology, University of California, San Francisco, United States; ^3^ Department of Pediatrics, University of California, San Francisco, San Francisco, CA, United States; ^4^ UCSF School of Medicine, University of California, San Francisco, United States; ^5^ Division of Pediatric Hematology/Oncology, Valley Children’s Hospital, Madera, CA, United States; ^6^ Department of Radiology and Biomedical Imaging, University of California, San Francisco, United States; ^7^ Children’s Hospital of Philadelphia, Philadelphia, PA, United States; ^8^ Department of Neurology, University of California, San Francisco, CA, United States; ^9^ Department of Neurological Surgery, University of California, San Francisco, CA, United States

**Keywords:** pediatric cancer survivors, Apo E4, neurocognition, late effects, radiation

## Abstract

**Background:**

Neurocognitive deficits in pediatric cancer survivors occur frequently; however, individual outcomes are unpredictable. We investigate clinical, genetic, and imaging predictors of neurocognition in pediatric cancer survivors, with a focus on survivors of central nervous system (CNS) tumors exposed to radiation.

**Methods:**

One hundred eighteen patients with benign or malignant cancers (median diagnosis age: 7; 32% embryonal CNS tumors) were selected from an existing multi-institutional cohort (RadART Pro) if they had: 1) neurocognitive evaluation; 2) available DNA; 3) standard imaging. Utilizing RadART Pro, we collected clinical history, genomic sequencing, CNS imaging, and neurocognitive outcomes. We performed single nucleotide polymorphism (SNP) genotyping for candidate genes associated with neurocognition: *COMT*, *BDNF*, *KIBRA*, *APOE*, *KLOTHO.* Longitudinal neurocognitive testing were performed using validated computer-based CogState batteries. The imaging cohort was made of patients with available iron-sensitive (n = 28) and/or T2 FLAIR (n = 41) sequences. Cerebral microbleeds (CMB) were identified using a semi-automated algorithm. Volume of T2 FLAIR white matter lesions (WML) was measured using an automated method based on a convolutional neural network. Summary statistics were performed for patient characteristics, neurocognitive assessments, and imaging. Linear mixed effects and hierarchical models assessed patient characteristics and SNP relationship with neurocognition over time. Nested case-control analysis was performed to compare candidate gene carriers to non-carriers.

**Results:**

CMB presence at baseline correlated with worse performance in 3 of 7 domains, including executive function. Higher baseline WML volumes correlated with worse performance in executive function and verbal learning. No candidate gene reliably predicted neurocognitive outcomes; however, *APOE ϵ4* carriers trended toward worse neurocognitive function over time compared to other candidate genes and carried the highest odds of low neurocognitive performance across all domains (odds ratio 2.85, *P*=0.002). Hydrocephalus and seizures at diagnosis were the clinical characteristics most frequently associated with worse performance in neurocognitive domains (5 of 7 domains). Overall, executive function and verbal learning were the most frequently negatively impacted neurocognitive domains.

**Conclusion:**

Presence of CMB, *APOE ϵ4* carrier status, hydrocephalus, and seizures correlate with worse neurocognitive outcomes in pediatric cancer survivors, enriched with CNS tumors exposed to radiation. Ongoing research is underway to verify trends in larger cohorts.

## Introduction

Pediatric cancer survivors, particularly survivors of central nervous system (CNS) tumors, suffer from a range of late effects related to their tumor diagnosis and therapies, often leading to long-term negative impacts on quality of life (1, 2). Arguably, one of the most challenging late effects seen in this population is neurocognitive impairment (3–5). This is especially true after exposure to CNS radiation. Adults that survive childhood CNS tumors have lower intelligence quotients (IQs) and neurocognitive deficits specific to a variety of domains such as attention, processing speed, and executive function that worsen over time (6–10). Although neurocognitive outcomes for pediatric CNS tumor survivors show poorer neurocognitive functioning when compared to population means and normal matched controls (4, 11, 12), there remains great variability among individuals (7, 13). It is well supported that certain interventions such as cranial radiation therapy (14), particularly in the youngest patients, negatively impact neurocognition. Other clinical characteristics such as young age, hydrocephalus, and seizure disorder at diagnosis have also shown inverse relationships with later neurocognitive aptitude (15, 16).

Across adult literature, limited pediatric literature, and in preclinical models for aging and dementia, there are several candidate genes linked to neurocognitive outcomes. Within these genes, there are single nucleotide polymorphisms (SNPs) that correlate with neurocognitive performance. In aging adult populations, catechol-O-methyltransferase (*COMT*, rs4680); brain-derived neurotrophic factor (*BDNF*, rs6265); kidney and brain expressed protein (*KIBRA*, rs17070145); apolipoprotein E (*APOE*, rs429358, rs7412); and klotho (*KL*, rs9536314, rs95270025) each carry allelic variants that can be beneficial or detrimental to neurocognition (17–19). *APOE*, *BDNF*, and *COMT* candidate genes have demonstrated influence on neurocognition in oncology populations, though limited data exists specific to pediatric cancer and CNS tumor populations (20, 21). Confirmation of the role of such genetic predictors on neurocognitive outcomes in pediatric cancer survivors, particularly those with CNS tumors, could help personalize cancer therapy with the potential to limit neurocognitive injury and refine follow-up care. Further, at diagnosis, identification of predictors would help families make treatment-related decisions; prepare families for potentially significant, long-term impacts on their child’s life; and identify children at greatest risk.

In addition to genetic correlates, radiographic and radiogenomic signatures of neurocognitive outcomes would augment our understanding of which patients are at greatest risk of neurocognitive injury and who may benefit from early educational or cognitive interventions. Patients who undergo cranial radiation are at risk of developing cerebral microbleeds (CMB), which associate with higher doses of radiation, volume of radiation field, longer follow up, and age (22–26). High resolution 7T MRI studies have reported as high as 100% prevalence in CMB detection after 1 year following radiation therapy (27, 28) CMB presence in the frontal lobe associates with worse performance in executive functioning in the RadART cohort (29). Similarly, white matter lesions (WML), as measured by T2 FLAIR sequences on MR imaging, are an established neuroimaging marker of chronic effects of pediatric cancer therapy, such as radiation. The risk of accumulating WMLs is increased by younger age at diagnosis, hydrocephalus, methotrexate exposure, and treatment with radiation. Further, higher dose and volume of irradiated tissue impact the accumulation of WMLs (22, 23, 25, 30), which ultimately correlate with negative effects on intelligent quotient (IQ) and cognitive domains such as processing speed (31, 32).

In the current study, we assessed the impact of clinical characteristics, CMB and WML, and cognition-related genes (*COMT*, *BDNF*, *KIBRA*, *APOE*, and *KLOTHO*) on neurocognitive outcomes in a cohort of pediatric cancer survivors, enriched with CNS tumors, using an established multi-institutional cohort (Rad ART Pro) (29, 33). We hypothesize certain clinical characteristics, extent of CMB and WML, and genetic variants related to cognition will augment prediction of neurocognitive outcomes in survivors of CNS tumors. Our long-term aim is to improve anticipatory guidance, contribute to treatment stratification, and improve protective interventions for this high-risk population.

## Methods

### Patient Population

The patient population included in this study was selected from a cohort of patients who were previously enrolled in a multicenter, longitudinal cohort investigating radiation-induced arteriopathy, RadART Pro. The study collects clinical characteristics, DNA samples from peripheral blood collections, imaging, and neurocognitive performance outcomes in pediatric cancer survivors (29, 33). The cohort is enriched with patients with CNS tumors previously exposed to radiation therapy. Initial inclusion criteria for enrollment into RadART Pro were: 1) prior diagnosis of cancer, 2) previous exposure to radiation of the brain and/or neck, 3) age ≤ 21 years at time of radiation exposure, 4) anticipated survival > 1 year post-radiation. In 2015, the study expanded to include a comparison group of pediatric brain tumor patients that did not receive radiation therapy. For this group, diagnosis of a brain tumor must have occurred at age ≤ 21 years. Patients were recruited from four sites, including UCSF Benioff Children’s Hospital – San Francisco and Oakland sites (San Francisco, CA; Oakland, CA); Valley Children’s Hospital (Madera, CA); and St, Louis Children’s Hospital (St. Louis, MO). The institutional review boards of all participating sites of the RadART Pro study approved the protocol and procedures for that study. Informed consent was obtained from all patients prior to participation.

To be included in the integrated analyses in the current work, patients must have had at least candidate gene sequencing and one timepoint of neurocognitive testing.

### Genotyping

SNP genotyping for *COMT*, *BDNF*, *KIBRA*, *APOE*, and *KLOTHO* was performed for each patient (**Supplemental Table 1**). Amplified product was sequenced in both directions with PCR primers using the Sanger method (Quintara Biosciences, Berkeley, CA). The complete sequencing protocol is included in the **Supplemental Methods** section.

### Neurocognitive Testing

Neurocognitive assessments were completed for all patients at an initial visit and regular follow-up intervals (about yearly) using computer-based CogState testing. CogState has been validated for patients 5 years and older, across a variety of populations, including the CNS tumor population (34–36). The CogState battery used in our study included the following tests: Identification test (IDN; attention), Continuous Paired Associate Learning test (CPAL; paired associate learning), Detection test (DET; psychomotor function), Groton Maze Learning test (GML; executive function), International Shopping List test (ISL and ISRL; verbal learning and verbal memory), and One Back test (ONB; working memory). All tests were administered by trained clinical research associates during standard of care clinic visits and under appropriate test-taking environments. Scores were collected for each test and converted to z-scores based on age-normed population means. For younger ages, some tests lacked sufficient population norms (e.g. age 5 to 9 years for the Groton Maze Learning, International Shopping List, and Continuous Paired Associate Learning tests). In these instances, z-scores were derived from age-matched comparisons within the patient cohort itself, as per vendor guidance.

Initial neurocognitive screens were typically conducted following completion of tumor-directed therapy for the primary diagnosis. Subsequent screens were completed at standard of care clinic visits for regular tumor surveillance. Due to the nature of this study opening several years after some patients completed therapy, initial testing occurred at variable post-therapy time points for individual patients. A continuous variable, “time from radiation” was used in all models to address the heterogeneity in timing of initial testing and follow-up. This variable reflected the time in years from end of cranial radiation therapy to the follow-up time point being tested.

### Imaging

Participants enrolled in RadART Pro were followed prospectively with structural and cerebrovascular brain imaging, as available. Imaging interval and acquisition parameters were based on institutional standards for routine clinical care and tumor surveillance. All imaging was performed on 1.5 and 3T scanners. Iron-sensitive imaging and T2 FLAIR sequences were acquired following primary therapy completion and within 180 days of the date of neurocognitive assessment.

Iron-sensitive imaging including T2*-weighted gradient echo sequences or susceptibility-weighted imaging (SWI; a technique that combines T2* magnitude and phase images to further enhance susceptibility contrast) were collected to detect, segment, and quantify CMBs using MATLAB-based semi-automated CMB detection and segmentation (37). CMBs were defined as hypointense foci that were present on consecutive, axial slices exceeding a threshold degree of radial symmetry (38). CMB candidates were excluded if in close proximity to perpendicular vessels or the tumor cavity. A single reader (LB) reviewed CMB candidates to determine if the segmented lesions were true CMBs or false positives. Segmented CMBs were counted and the cumulative CMB aggregate volume (mm^3^) was calculated.

T2-weighted fluid-attenuated inversion-recovery (FLAIR) sequences were collected across study sites, with inter-site variability in two-dimensional and three-dimensional acquisitions. A previously described convolutional neural network with 3D U-net architecture (39, 40) was trained to identify abnormal FLAIR signal attributable to prior radiation, excluding abnormal FLAIR signal attributable to post-surgical changes or treated tumor tissue. Training data consisted of 246 expert manual segmentations of target FLAIR signal, which were initially segmented by a research specialist with several years of brain MRI segmentation experience and modified or verified by a board-certified neuroradiologist with 4 years of post-residency experience. Training data were independent of test data, noting that 9 of the MRIs used for training were from patients that were also included in the test set, but from MRIs obtained in different years from those in the test set. Training hyperparameters included a kernel size of 3 x 3 x 3, cross-entropy loss function, and an Adam optimizer with learning rate of 1 x 10−4, implemented in TensorFlow 2 (https://www.tensorflow.org) using the Python programming language. The network was trained for 110 epochs, with a batch size of 37 3D patches (96 x 96 x 96 mm each). The implementation was on a DGX-2 AI server (version 4.5.0; NVIDIA). The fully trained U-net was then applied to the patients in our cohort with available FLAIR sequences and neurocognitive assessments to detect and segment areas of abnormal FLAIR signal attributable to radiation treatment, and the volume of this abnormality was quantified.

### Statistical Analysis

Clinical, genomic, and imaging variables were defined as follows: time from radiation (continuous variable), age at diagnosis (continuous variable), presence of hydrocephalus at diagnosis (binary variable), presence of seizures at diagnosis (binary variable), chemotherapy exposure (binary variable), radiation exposure (binary variable), gender (binary variable), tumor type (categorical variable), tumor location (categorical variable), presence of CMB at baseline (binary variable), and WML volume at baseline (continuous variable). Radiation was included as a binary variable to accommodate patients for which we did not have details on radiation dose. Across each model and statistical comparison, neurocognitive outcomes were evaluated per neurocognitive domain tested.

Summary statistics for patient characteristics, neurocognitive assessments, and imaging variables are presented as frequencies and percentages for categorical measures and median and interquartile range (IQR) for continuous variables. Neurocognitive outcomes are plotted over time with trajectories stratified by SNP carrier status (heterozygous or homozygous [carrier] vs non-carrier). Linear mixed effects models with time from radiation were used to assess the significance of SNP carriers on neurocognitive outcomes over time. The association of patient characteristics on neurocognitive outcomes were evaluated similarly. Characteristics significantly associated with most neurocognitive assessments were included in adjusted models considering SNP carrier status effect on outcome measures. Baseline association of imaging variables with patient characteristics, neurocognitive assessments, and SNP carrier status were evaluated by Chi-Square or Fisher’s exact tests, Wilcoxon rank-sum or Kruskal-Wallis tests, or Spearman correlation as appropriate. Hierarchical modeling with the addition of baseline CMB presence and WML volume to our adjusted models is used to assess longitudinal effect. All inference was conducted with significance level 0.05. All analyses were figures are generated in R 4.1.2.

Nested case-control analysis was done to compare candidate gene carriers (cases) and non-carriers (controls). Odds ratios were calculated to compare the prevalence of carriers and non-carriers in the lowest and highest performers on neurocognitive testing. Scores that were at least one standard deviation above or below the mean were considered high and low performers, respectively.

## Results

### Cohort Descriptions

#### Overall Cohort

**Figure 1:**
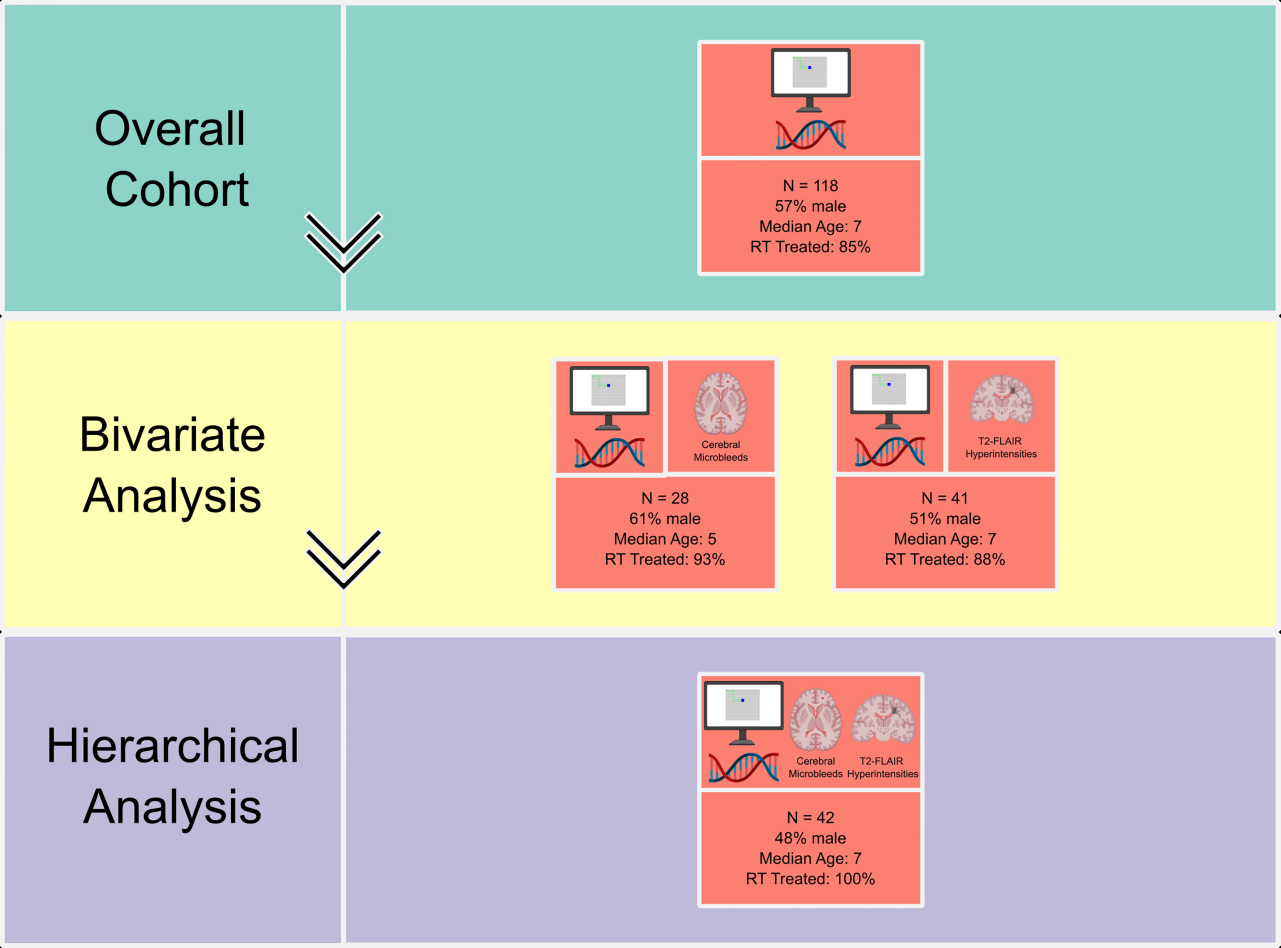
Diagram of modalities investigated and bivariate and multivariate analyses with individual subcohort size and characteristics. Diagram details delineate data type at each level of analysis: neurocognitive assessments (computer screen), candidate gene sequencing (double helix), and imaging (CMBs as axial view, FLAIR WML as coronal view). RT, radiation therapy. Created with BioRender.com.

Within the full RadART Pro cohort (n=447), 118 patients met criteria for completion of both candidate sequencing and at least one timepoint of neurocognitive testing (n=57 males; median age at diagnosis 7 years [IQR 4, 11]; **Table 1** and **Figure 1**). A total of 28 patients in this cohort had available iron-sensitive imaging sequences for assessment of CMBs and 41 patients had T2 FLAIR imaging for assessment of WMLs. These subcohorts are described in detail below.

**Table 1:** Summary of patient demographics, tumor characteristics, and baseline clinical symptoms across each subcohort by column.

Characteristics	Overall (n=118)	CMBs (n=28)	White matter changes (n=41)
**Age at diagnosis, years (median [IQR])**			
**Diagnosis**	7 (4, 11)	5.0 (3, 8)	7 (3, 10)
**Gender, n (%)**			
**Male**	67 (57)	17 (61)	21 (51)
**Race, n (%)**			
American Indian or Alaska Native	1 (1)	0 (0)	0 (0)
Asian	12 (10)	3 (11)	7 (17)
Black or African American	4 (3)	1 (4)	2 (5)
Multiracial	5 (4)	3 (11)	0 (0)
Native Hawaiian or Other Pacific Islander	1 (1)	0 (0)	0 (0)
Unknown	13 (11)	2 (7)	3 (7)
White	82 (70)	19 (68)	29 (71)
**Ethnicity, n (%)**			
Hispanic or Latino	30 (25)	2 (7)	8 (20)
Not Hispanic or Latino	88 (75)	26 (93)	33 (80)
**Tumor Type, n (%)**			
Embryonal tumors	38 (32)	11 (39)	19 (46)
Hematologic Malignancy	21 (18)	0 (0)	1 (2)
Low-grade glioma	16 (14)	6 (21)	6 (15)
NOS	13 (11)	4 (14)	5 (12)
Ependymal tumors	8 (7)	1 (4)	4 (10)
Solid tumors (extra-CNS)	8 (7)	0 (0)	0 (0)
Germ cell tumors	7 (6)	2 (7)	5 (12)
High-grade glioma	7 (6)	4 (14)	1 (2)
Tumor Location, n (%)			
Cerebellum/Posterior fossa	35 (30)	9 (32)	17 (41)
Extra-CNS	31 (26)	0 (0)	1 (2)
Midline	21 (18)	8 (29)	12 (29)
Lobar	14 (12)	6 (21)	4 (10)
NOS	6 (5)	2 (7)	1 (2)
Multifocal	5 (4)	2 (7)	3 (7)
Optic nerves	5 (4)	1 (4)	3 (7)
**Radiation therapy exposure, n (%)**	100 (85)	26 (93)	36 (88)
**Hydrocephalus at diagnosis, n (%)**	40 (34)	13 (46)	18 (44)
**Seizures at diagnosis, n (%)**	14 (12)	2 (7)	6 (15)

Details of each cohort are provided, including demographics of patients and diagnoses and treatment details. CMBs, cerebral microbleeds; IQR, interquartile range; WMLs, white matter lesions; NOS, not otherwise specified.

Embryonal tumors were the most frequent tumor diagnosis (n=38, 32%) with cerebellum/posterior fossa being the most common primary tumor location (n=35, 30%). Most patients (n=100, 85%) were treated with radiation therapy. Median time from cranial radiation therapy to time of initial neurocognitive testing was 3.9 years (IQR 2.1, 6.5) and median age at time of initial neurocognitive testing was 13 years (IQR 9.0, 18). Median time from diagnosis to initial neurocognitive testing was 5.0 years (IQR 3.0, 8.0; **Table 2**).

**Table 2:** Time of initial neurocognitive assessments in relationship to patient diagnosis and radiation by subcohort.

Temporal characteristics at baseline Cogstate testing	Overall Cohort	CMBs	WMLs
	n = 118	n = 28	n = 41
Age, years [median (IQR)]	13 (9, 18)	13 (9,15)	12 (9, 17)
Time from diagnosis, years [median (IQR)]	5.0 (3.0, 8.0)	6.5 (4.0, 9.0)	6.0 (3.0, 8.0)
Time from radiation therapy, years [median (IQR)]	3.9 (2.1, 6.5)	4.5 (2.5, 6.5)	4.3 (1.5, 6.4)

Table describes age at time of Cogstate neurocognitive testing, time from diagnosis to testing, and time from radiation to testing. IQR, interquartile range; CMBs, cerebral microbleeds; WMLs, white matter lesions.

#### CMB Subcohort

**Figure 2:**
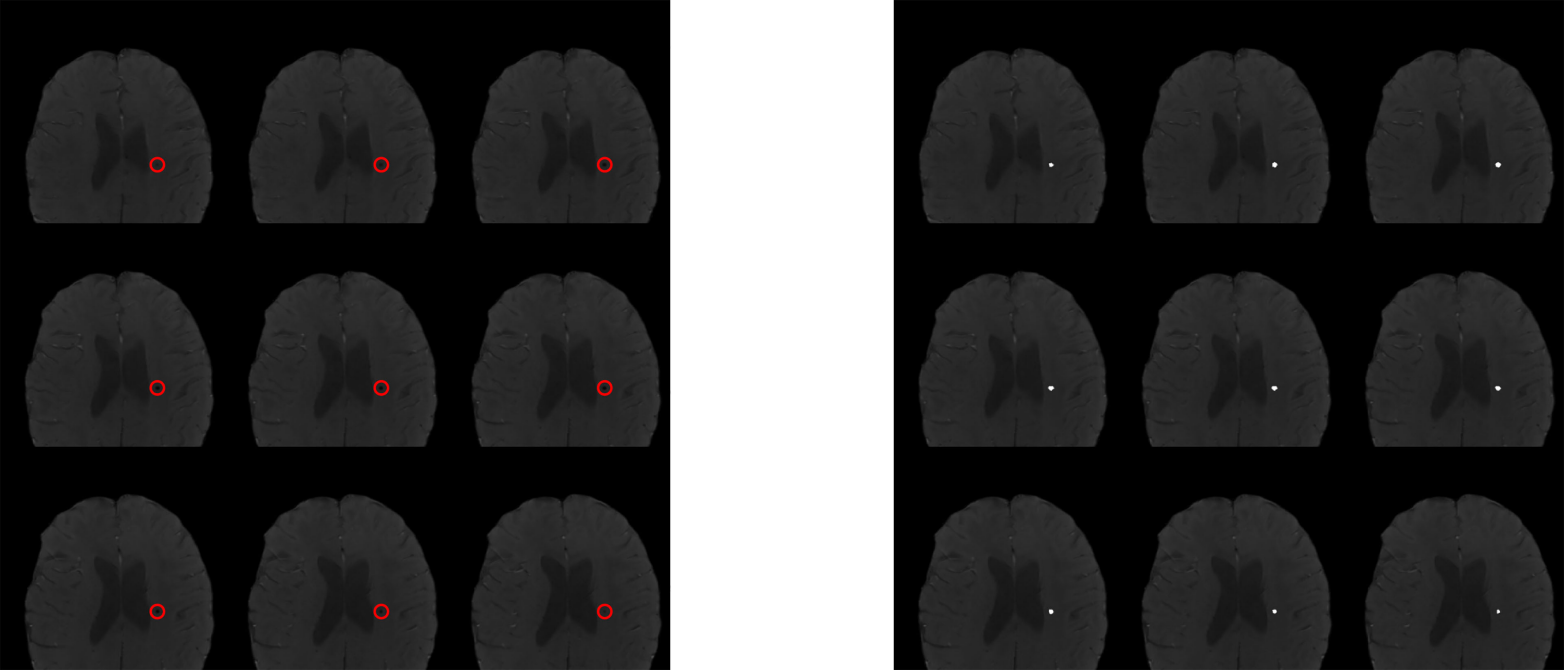
Visual representation of CMB analysis. Imaging inclusive of semi-automated lesion segmentation iron-sensitive sequence analysis. Left panel shows sequence without segmentation label with manually insertion of red circle outlining area of known cerebral microbleed. Right panel displays with semi-automated insertion of white circle overlying area of cerebral microbleed identified on segmentation.

Of the 28 patients with available iron-sensitive imaging, 17 were male (61%; median age of diagnosis was 5 years [IQR 3, 8]; **Table 1**). Embryonal tumors were the most frequent tumor diagnosis (n = 11, 39%) with cerebellum/posterior fossa as the most common primary tumor location (n=9, 32%). Most patients (n=26, 93%) were treated with radiation therapy. Median age at initial neurocognitive assessment was 13 years (IQR 9, 15) and median time from diagnosis to initial neurocognitive testing was 6.5 (IQR 4.0, 9.0; **Table 2**). In patients previously treated with radiation therapy, median time from radiation therapy to initial neurocognitive testing was 4.5 years (IQR 2.5, 6.5). At least one CMB was detected in 10 patients (36%) at the time of initial neurocognitive assessment. Among those with at least one CMB, the median number of CMBs was 5.0 (IQR 4.0, 5.0) and the median total volume of CMBs was 120 mm^3^ (**Figure 2**). Neither the age at diagnosis nor the time from radiation significantly differed between the CMB-present and CMB-absent groups.

#### WML Subcohort

**Figure 3:**
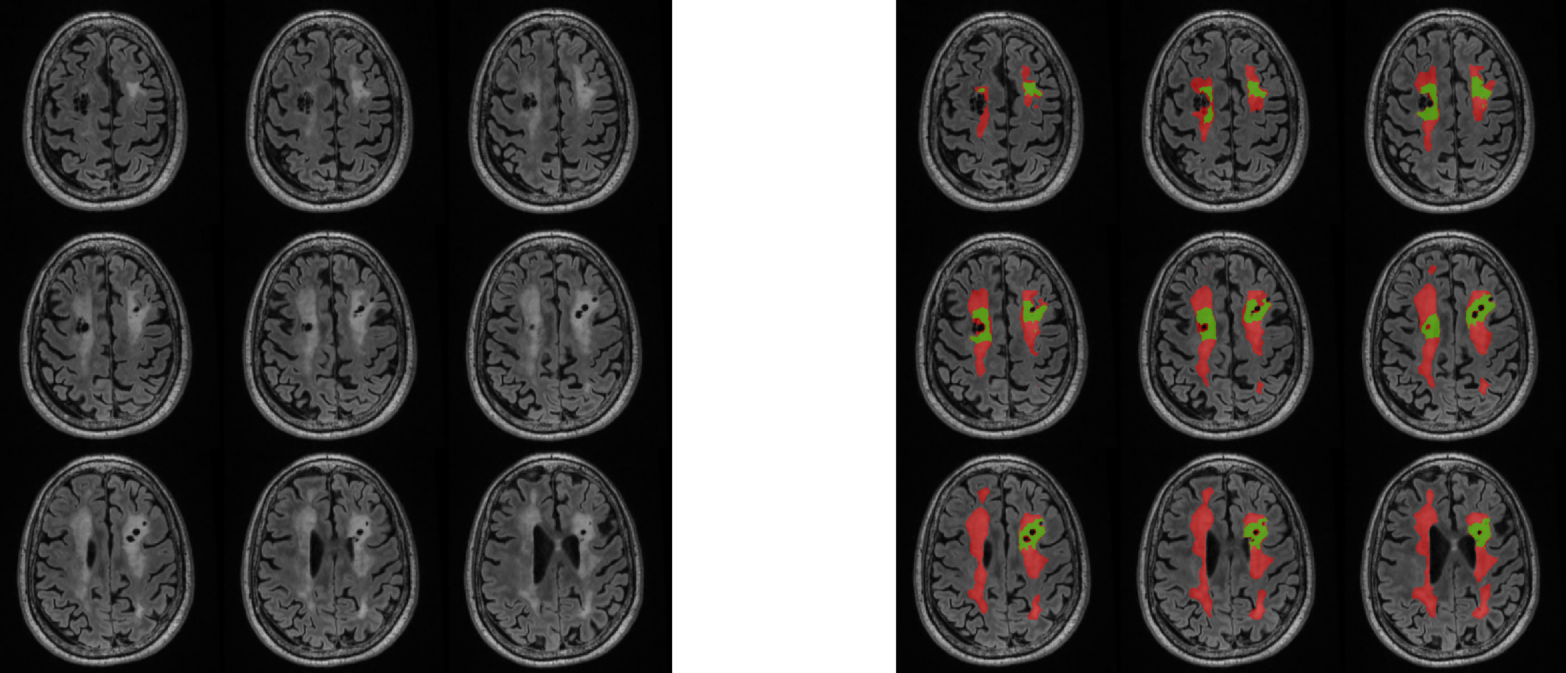
Visual representation of WML analysis. Imaging inclusive of manual T2-FLAIR white matter lesion segmentations with RT-induced (red) and non-RT-induced (green) labeling. RT, radiation therapy.

Of the 41 patients with available T2 FLAIR imaging, 21 were male (51%; median age of diagnosis was 7 years [IQR 3. 10]; **Table 1**). Embryonal tumors were the most frequent tumor diagnosis (n = 19, 46%) with cerebellum/posterior fossa as the most common primary tumor location (n=17, 41%). Most patients (n=36, 88%) were treated with radiation therapy. Median age at initial neurocognitive assessment was 12 years (IQR 9, 17 and median time from diagnosis to initial neurocognitive testing was 6.0 years (IQR 3.0, 8.0; **Table 2**). In patients previously treated with radiation therapy, median time from radiation therapy to initial neurocognitive testing was 4.3 years (IQR 1.5, 6.4). Most patients (n=39; 95%) had measurable WML volumes identified by the convolutional neural network, of which the median volume was 1400 mm^3^ (IQR 349, 4590; **Figure 3**).

### Prevalence of Candidate Genes

Each genetic variant was present at varying prevalence across the cohorts, with the largest difference in candidate gene carrier proportion being 32% and 46% for BDNF rs6265 in the CMB and WML cohorts, respectively (**Supplemental Table 2**). For the subset of 118 total patients, sequencing was unsuccessful for individual two alleles of interest (*APOE*: n=9; *BDNF*: n=1).

### Clinical, Genomic, and Imaging Effects on Neurocognitive Outcomes

We initially performed bivariate analyses to identify isolated associations between clinical, genomic, and imaging characteristics with neurocognitive outcomes and inclusive of time to radiation as a variable. Based on statistical significance in bivariate analyses, we then determined which variables would be used in a hierarchical analysis to identify contributions of multiple variables on each neurocognitive domain tested.

#### Clinical characteristics

Hydrocephalus and seizures were the most common clinical characteristics associated with worse neurocognitive outcome. These were each associated with 5 of 7 domains tested, including verbal learning, verbal memory, working memory, attention, and executive function (**Table 3**). Of tumor diagnoses, germ cell tumors associated with the highest number of affected domains (3 of 7), including verbal learning, working memory, and executive function. Age at diagnosis associated with worse performance in attention (*P*=0.03) and time from radiation associated with worse performance in verbal learning (*P*=0.03).

**Table 3:** Patient and imaging characteristics associated with domains of neurocognitive outcomes.

Neurocognitive Domain	Hydrocephalus	Seizures	Time from RT	CMBs	WML volume
	n = 118	n = 118	n =100	n= 28	n = 41
Executive functioning (GML)	(0.05)	0.0009	0.16	0.02	(0.05)
Verbal learning (ISL)	0.0002	0.003	0.03	0.03	0.06
Working memory (ONB)	0.0005	0.03	0.85	0.13	0.31
Attention (IDN)	0.02	0.01	0.89	0.13	0.49
Verbal memory (ISRL)	0.0001	0.002	0.39	0.10	0.77
Psychomotor functioning (DET)	(0.05)	0.19	0.30	0.01	0.51
Paired associate learning (CPAL)	0.15	0.76	0.77	0.36	0.19

Patient clinical characteristics, baseline CMB, and baseline WML volume (top row) associations with neurocognitive outcomes (left column) in bivariate analysis with inclusion of time from radiation. Cells contain statistically significant P-values (P < 0.05). Three comparisons reach borderline association indicated by parentheses (P = 0.05). Clinical characteristics analyzed and not displayed in table include age at diagnosis, chemotherapy exposure, and tumor location. RT, radiation therapy; CMBs, cerebral microbleeds; WML, white matter lesion.

#### Genomic Characteristics

**Figure 4:**
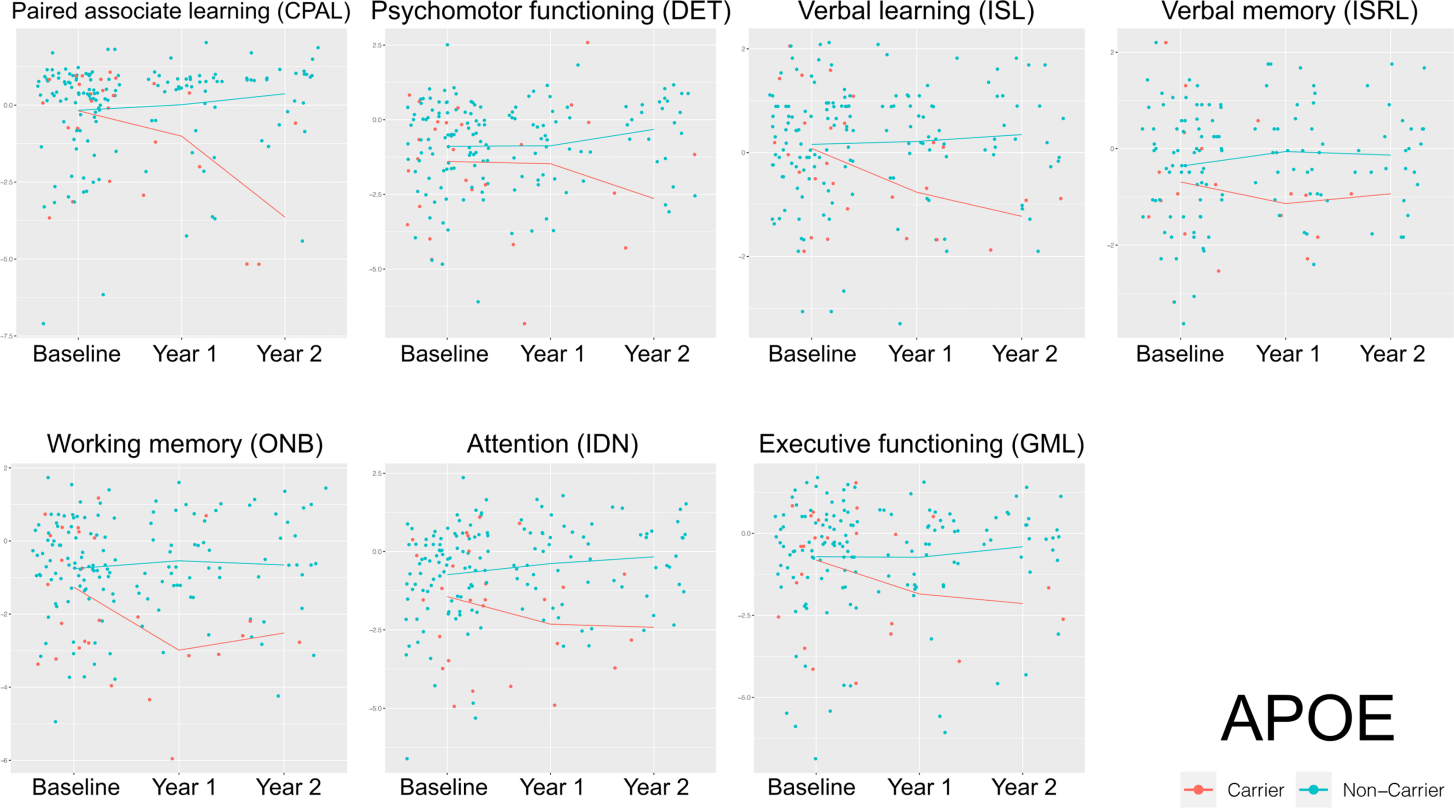
Longitudinal impact of *APOE ϵ4* carrier status across each neurocognitive domain tested at baseline, Year 1, and Year 2 of enrollment. Trajectory of *APOE ϵ4* carrier versus non-carrier mean performance across each neurocognitive domain from initial neurocognitive testing (baseline) to timepoint 3 of neurocognitive testing (Year 2). Blue line=non-carrier, red line=carrier.

No candidate gene reliably predicted neurocognitive outcomes at a statistically significant level. However, compared to non-carriers, *APOE ϵ4* carriers demonstrated worsening neurocognitive performance over time across all domains, albeit in a small cohort in later years of analysis (**Figure 4**). No other genetic variants demonstrated obvious trends on neurocognitive outcomes over time (**Supplemental Figures 1**–**4**). In the nested case-control candidate gene analyses comparing proportion of *APOE ϵ4 carrier and non-carriers within the highest and lowest performers in neurocognitive testing*, *APOE ϵ4* carriers had the greatest odds of being among the poorest performers across all neurocognitive domains at all time points tested (Odds ratio [OR] 2.85, *P*=0.002), *BDNF* carriers showed the lowest odds, (OR 0.52, *P*=0.001), and *COMT* did not reach statistical significance (**Table 4**).

**Table 4:** Prevalence of candidate gene carriers among high and low performers of neurocognitive assessments for timepoints 1 to 3 of testing.

Candidate gene (allele of interest)	High performers, n (% overall)	Low performers, n (% overall)	Odds ratio (95% CI)	*P*-Value
***APOE***	11 (6)	75 (43)	2.85 (1.46, 5.57)	0.002
***BDNF***	69 (13)	127 (24)	0.53 (0.36, 0.78)	0.001
***COMT***	79 (10)	241 (30)	1.31 (0.90, 1.90)	0.16
***KIBRA***	103 (10)	328 (33)	1.97 (1.28, 3.05)	0.002
***KLOTHO***	19 (6)	107 (34)	2.47 (1.45, 4.20)	0.0008

Candidate gene alleles of interest (left column) carrier status with associated prevalence among high (>1 SD from mean) and low performers (<1 SD from mean) across any neurocognitive domain at all timepoints of testing. Odds ratio with 95% confidence interval indicates increased (APOE, KIBRA, KLOTHO) versus decreased risk (BDNF) of being among low performers. Last column contains statistically significant P-value (P < 0.05).

No candidate gene was found to correlate with CMBs or WML volume at baseline assessment.

#### Imaging Characteristics

The impact of baseline CMBs and WML volume on neurocognition was evaluated independently and in combination. The presence of CMBs at time of initial neurocognitive assessment associated with worse performance in psychomotor function, executive function, verbal learning with median z-scores across each domain of -1.84 (*P*=0.01), -1.75 (*P*=0.02), and -0.77 (*P*=0.03), respectively. The impact of volume of WMLs at time of initial neurocognitive assessment was evaluated using Spearman correlation coefficients. Higher baseline WML volumes trended with worse performance in executive function (*P*=0.05) and verbal learning (*P*=0.06), but these did not reach statistical significance.

#### Hierarchical Cluster Analysis

While we recognize limitations of our cohort size, we sought to preliminarily explore interactions of possible neurocognitive predictors. Based on statistical significance from bivariate analyses combined with prior work to suspect association with neurocognitive outcome (i.e. time from radiation), we performed hierarchical analyses across each candidate gene and neurocognitive outcome. The first model incorporated candidate gene carrier status, hydrocephalus, seizures, tumor type and time from radiation, and the second model added CMB and WML volume at baseline. Across the models without imaging findings, hydrocephalus or seizures at baseline continued to be the most prevalent characteristics associated with negative neurocognitive outcomes. No candidate gene demonstrated significant association once other characteristics were considered. Across these models, verbal learning, memory, working memory, executive function were consistently significantly impacted, most frequently in association with baseline hydrocephalus or seizures (*P*<0.05). In contrast, paired associate learning, psychomotor function, and attention were not significantly impacted across the variables tested.

## Discussion

As children complete therapy and enter long-term surveillance, identification of those at high-risk of neurocognitive injury based on genomics and/or radiographic imaging could lead to more aggressive and earlier neurocognitive and educational intervention. Our study sought to broaden understanding of predictors of neurocognitive outcomes in pediatric cancer survivors. Compared to adult populations, few studies exist that investigate genetic predictors for neurocognition in long-term survivors (17, 18, 41–43). *COMT* has been investigated in childhood brain tumor survivors, where Met/Val heterozygotes outperform Met/Met and Val/Val homozygotes (rs4680; Val158Met) (44). And, a common polymorphism in *BDNF*, Val66Met, shows valine homozygosity associates with higher IQs, processing speed, and memory in adults (17), but lacks demonstrated impact on neurocognitive function in adult CNS tumor survivors (20). In other adult studies, heterozygosity for the *KL* haplotype, KL-VS (Phe352Val and Cys370Ser), leads to improved cognition, executive function, and larger brain volumes in aging adults and transgenic mouse models (18, 43, 45). Meanwhile, in healthy children, *APOE ϵ4* homozygotes have shown poorer executive function, memory, and attention, and a potential relationship to smaller hippocampal volumes (46). Further, *APOE ϵ4* has been linked to poorer neurocognition, memory, and executive function in adult CNS tumor patients (19, 47, 48) and was linked to tau-mediated neuroinflammation and neurodegeneration, independent of amyloid-ß deposition (19, 49).

Although no single gene was a reliable predictor across all tested neurocognitive domains in our cohort, *APOE ϵ4* carrier status most robustly associated with neurocognitive worsening over time. Other candidate genes, *COMT, BDNF, KIBRA* and *KLOTHO*, demonstrated mixed impact on neurocognitive outcomes and sometimes unexpected impact, such as the apparent protective effect of *BDNF* Val66Met. We recognize that we did not explore all possible SNP possibilities that may play a role across these genes and explored only SNPs of interest, which may inform future analyses of this cohort. Further, the cohort of patients with the SNPs of interest remained small overall and warrants study in a larger population. Additionally, we did not fully explore circulating peripheral protein levels, which may inform genotype: phenotype relationships. For example, high circulating levels of BDNF negatively correlate with neurocognitive function (50), while elevated klotho levels correlate with improved cognitive (43, 45). A next iteration of our work will be to correlate protein levels with genotype, which is currently under way for cerebrospinal and blood collections from our cohort.

We previously demonstrated that the presence of CMBs is associated with poor neurocognitive outcomes in pediatric CNS tumor survivors (28, 29). Of particular interest to the current work is that CMBs also associate with Alzheimer’s disease (29, 51) and *APOE ϵ4* has been linked directly to CMBs in non-demented elderly patients, as well as neurovascular disease, decreased neuronal repair, and increased brain atrophy (52, 53). Given the role of *APOE ϵ4* across broad pathophysiologic processes, it is possible that radiation injury in the setting of *APOE ϵ4* exacerbates underlying predisposition to multifactorial neurocognitive injury. This would support our preliminary *APOE ϵ4* signal as a biomarker of neurocognitive outcomes in our patient cohort, which again was enriched with patients that previously received CNS radiation. Although our findings include a limited sample size, we feel they confirm that future exploration should explore volumetric analyses, white matter changes based on DTI sequences, and in larger patient numbers.

From a clinical standpoint, our work supported previous reports identifying hydrocephalus and seizures at baseline as predictors of worse cognitive function (2, 6, 8, 54–58). In our cohort, across both bivariate and multivariate analyses, hydrocephalus and seizures reliably correlated with worse performance across several domains, including executive function. Of interest was that age at diagnosis and time from radiation did not reliably correlate with worse outcomes as in Morrison et al. (28), while germ cell tumors seem to most commonly correlated with worse outcomes albeit in overall small numbers. The lack of impact from age and time could be in part due distinctions in analyses, including our use of age as a continuous variable compared to other analyses using age cut-offs as binary or categorical variables (56, 59–61). Additionally, from a longitudinal perspective, it is possible that because our patients were commonly already five or more years from diagnosis, we see a decreased impact on rate of change (i.e. patients entered the study at an already lower baseline performance). In contrast, the contribution of the germ cell diagnosis could be reflective of the radiation field in these tumors and overlap with critical structures like the hippocampus (12, 57–59), but the validity of finding related to germ cell tumors require verification in larger patient numbers.

The strengths of our study arise from the diversity of the patient population and long-term follow-up, typically spanning over five years. Our cohort included several different tumor types and tumor locations, as well as patients across a range of ages and demographics. By pooling of patients from four separate sites across California and Missouri, we capture a broad and diverse patient population from multiple socioeconomic backgrounds, races, and ethnicities. The longitudinal time points of follow-up also strengthen our findings, as neurocognitive injury in pediatric brain tumor survivors worsens over time and becomes more impacting as patients age (2, 62). Lastly, the consistency of the neurocognitive measurements and analyses among each gene solidified the comparison between genes, more so than if the genes had been evaluated in separate studies. In contrast, the main shortfalls of our study arise from non-standardized follow-up periods, late timepoints to initial neurocognitive testing, and overall small cohort size for multivariate analyses. First, there was a limited number of patients that had reliable follow-up and a cohort of patients with missing treatment details. This was both a result of not yet reaching the assigned time point of follow up, but also due to patients being lost to follow-up and in recent years and clinical research restrictions imposed by the COVID-19 pandemic. Unfortunately, this contributed to missingness within the hierarchical analysis, inclusive of imaging variables, and thus limited these models. Future studies will need to expand the cohort size to accommodate the number of variables. Second, the follow-up intervals are not uniform throughout the cohort. This was driven by the neurocognitive tests being given as part of standard of care clinic visits and follow-up appointments being patient-dependent. We attempted to address this in our statistical analysis by including time from radiation as a continuous variable. Additionally, the patient population, although diverse, predominantly included patients with embryonal tumors reflecting the fact that medulloblastoma is the most common malignant pediatric CNS tumor. Further, we did not delineate dose of radiation or type of chemotherapy and only included these as binary variables in this initial review. Lastly, we recognize the nature of our investigation does not utilize reliable change indices as has been previously proposed for longitudinal neuropsychological testing (63) and the derivation of some normative values for the youngest patients within our cohort can be considered a shortcoming of this study, as this normalization may skew z-scores to higher performance than if compared to healthy populations alone. On the other hand, given that all testing was analyzed in the same manner across all genetic variants, we feel the reliability of the comparison across the genes remains intact and we would not expect the trend differences between the genes to be impacted by practiced learning or developmental changes within age groups over time.

In summary, our work shows that CMBs, WMLs, *APOE ϵ4* carrier status, hydrocephalus and/or seizures at baseline may serve as markers for long-term cognitive dysfunction in pediatric cancer survivors, especially in patients with CNS tumors previously treated with radiation. Work is actively underway to expand the preliminary findings in this report and to include additional retrospective and prospective genetic and imaging studies for this target population.

## Data Availability Statement

The raw data supporting the conclusions of this article will be made available by the authors, without undue reservation.

## Ethics Statement

The studies involving human participants were reviewed and approved by UCSF Benioff Children’s Hospital – San Francisco and Oakland sites (San Francisco, CA; Oakland, CA); Valley Children’s Hospital (Madera, CA); and Washington University School of Medicine, St, Louis (St. Louis, MO).Written informed consent to participate in this study was provided by all participants and/or participants' legal guardian.

## Author Contributions

CK, SM, HF contributed to conception and design of the study. DS recruited and enrolled participants on to the study. WF, SS performed the statistical analysis with review done by CK. LB, JL, MM, AR, PN performed imaging data analysis. CK, SS wrote first draft of the manuscript. JL, MM, AR, PN, WF contributed to specific sections of the manuscript. CK, SS, WF created all figures and tables. All authors reviewed and provided feedback to manuscript first draft. CK, SS completed the manuscript final revision. All authors contributed to the article and approved the submitted version.

## Funding

This work was supported by a donation from the LaRoche family (HF, SM).

## Conflict of Interest

The authors declare that the research was conducted in the absence of any commercial or financial relationships that could be construed as a potential conflict of interest.

## Publisher’s Note

All claims expressed in this article are solely those of the authors and do not necessarily represent those of their affiliated organizations, or those of the publisher, the editors and the reviewers. Any product that may be evaluated in this article, or claim that may be made by its manufacturer, is not guaranteed or endorsed by the publisher.

## Supplementary Material

Supplementary Figure 1 Longitudinal impact of *BDNF* carrier status across each neurocognitive domain tested at baseline, Year 1, and Year 2 of enrollment. Trajectory of *BDNF* carrier versus non-carrier performance across each neurocognitive domain from initial neurocognitive testing (baseline) to timepoint 3 of neurocognitive testing (Year 2). Blue line=non-carrier, red line=carrier.

Supplementary Figure 2 Longitudinal impact of *COMT* carrier status across each neurocognitive domain tested at baseline, Year 1, and Year 2 of enrollment. Trajectory of *COMT* carrier versus non-carrier performance across each neurocognitive domain from initial neurocognitive testing (baseline) to timepoint 3 of neurocognitive testing (Year 2). Blue line=non-carrier, red line=carrier.

Supplementary Figure 3 Longitudinal impact of *KIBRA* carrier status across each neurocognitive domain tested at baseline, Year 1, and Year 2 of enrollment. Trajectory of *KIBRA* carrier versus non-carrier performance across each neurocognitive domain from initial neurocognitive testing (baseline) to timepoint 3 of neurocognitive testing (Year 2). Blue line=non-carrier, red line=carrier.

Supplementary Figure 4 Longitudinal impact of *KL-VS* carrier status across each neurocognitive domain tested at baseline, Year 1, and Year 2 of enrollment. Trajectory of *KL-VS* carrier versus non-carrier performance across each neurocognitive domain from initial neurocognitive testing (baseline) to timepoint 3 of neurocognitive testing (Year 2). Blue line=non-carrier, red line=carrier.

Supplementary Table 1 Description of candidate gene SNPs of interest and anticipated impact on neurocognitive outcome. Candidate gene alleles sequenced and expected effects on neurocognitive outcomes

Supplementary Table 2 Prevalence of candidate gene SNPs of interest across each investigated subcohort. Candidate gene allele frequencies by cohort across each row.

## Abbreviations

APOE4, apolipoprotein E (*APOE*, rs429358, rs7412); BDNF, brain-derived neurotrophic factor (*BDNF*, rs6265); COMT, catechol-O-methyltransferase (*COMT*, rs4680); CMBs, cerebral microbleeds; KIBRA, kidney and brain expressed protein (*KIBRA*, rs17070145); WML, white matter lesions.
