# Supplementary figures and images for: An Integrated Analysis of Clinical, Genomic, and Imaging Features Reveals Predictors of Neurocognitive Outcomes in a Longitudinal Cohort of Pediatric Cancer Survivors, Enriched with CNS Tumors (Rad ART Pro)
Source: Front Oncol. 2022 Jun 23;12:874317. doi: 10.3389/fonc.2022.874317 (PMC9259981; doi:10.3389/fonc.2022.874317)

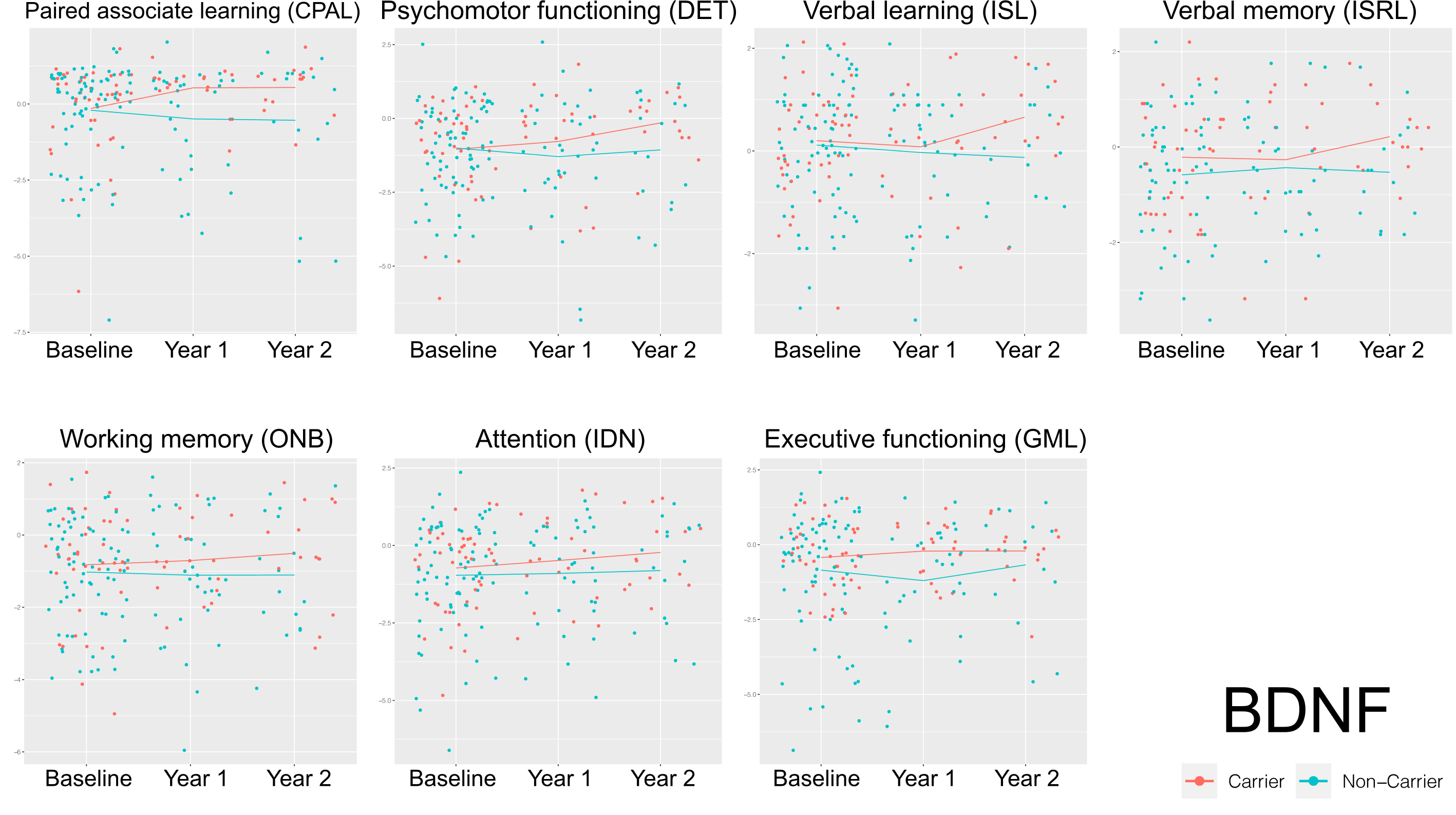

Supplement: Supplementary Figure 1 — Longitudinal impact of BDNF carrier status across each neurocognitive domain tested at baseline, Year 1, and Year 2 of enrollment. Trajectory of BDNF carrier versus non-carrier performance across each neurocognitive domain from initial neurocognitive testing (baseline) to timepoint 3 of neurocognitive testing (Year 2). Blue line=non-carrier, red line=carrier. [file Image_1.tiff]

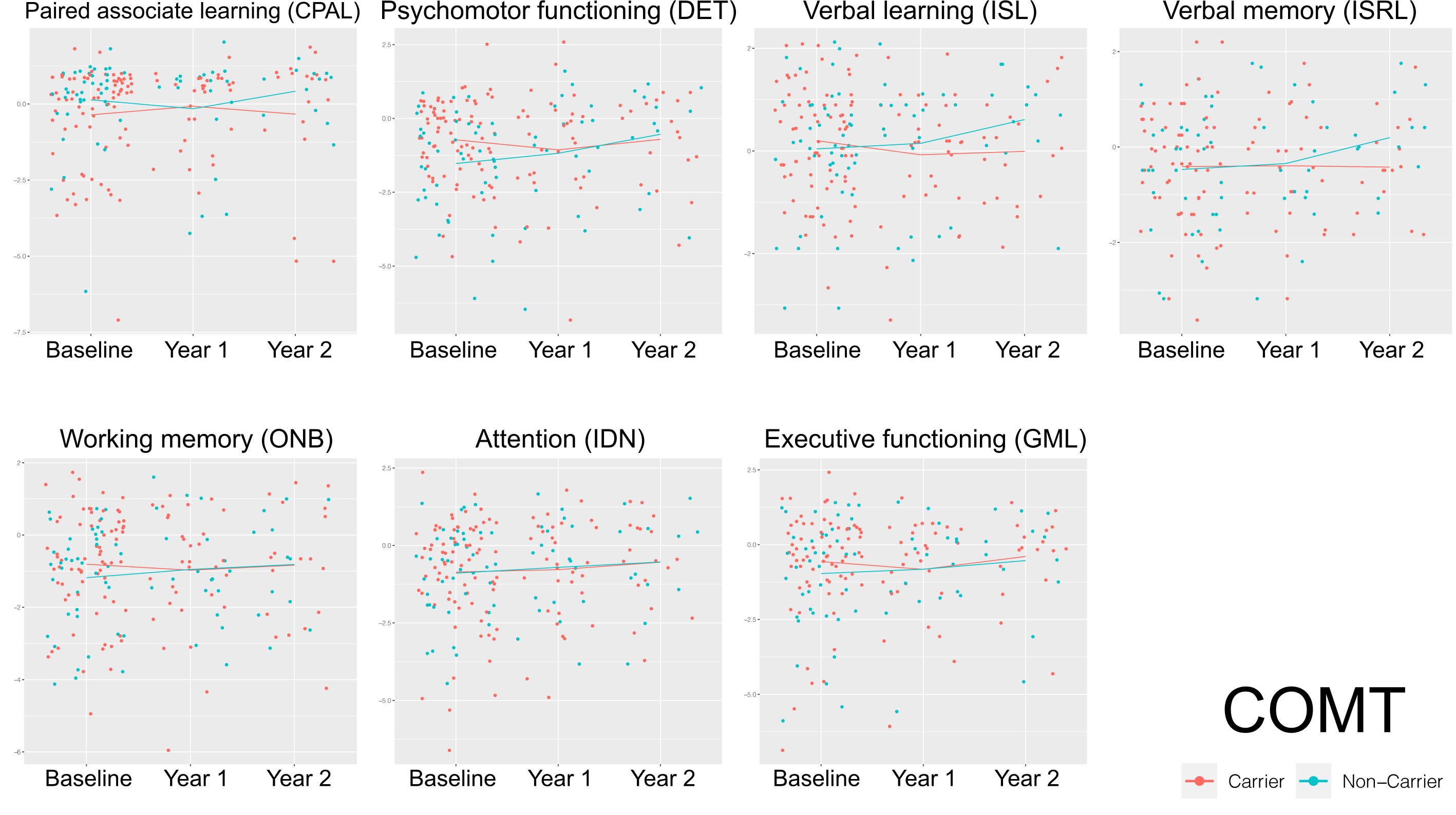

Supplement: Supplementary Figure 2 — Longitudinal impact of COMT carrier status across each neurocognitive domain tested at baseline, Year 1, and Year 2 of enrollment. Trajectory of COMT carrier versus non-carrier performance across each neurocognitive domain from initial neurocognitive testing (baseline) to timepoint 3 of neurocognitive testing (Year 2). Blue line=non-carrier, red line=carrier. [file Image_2.tiff]

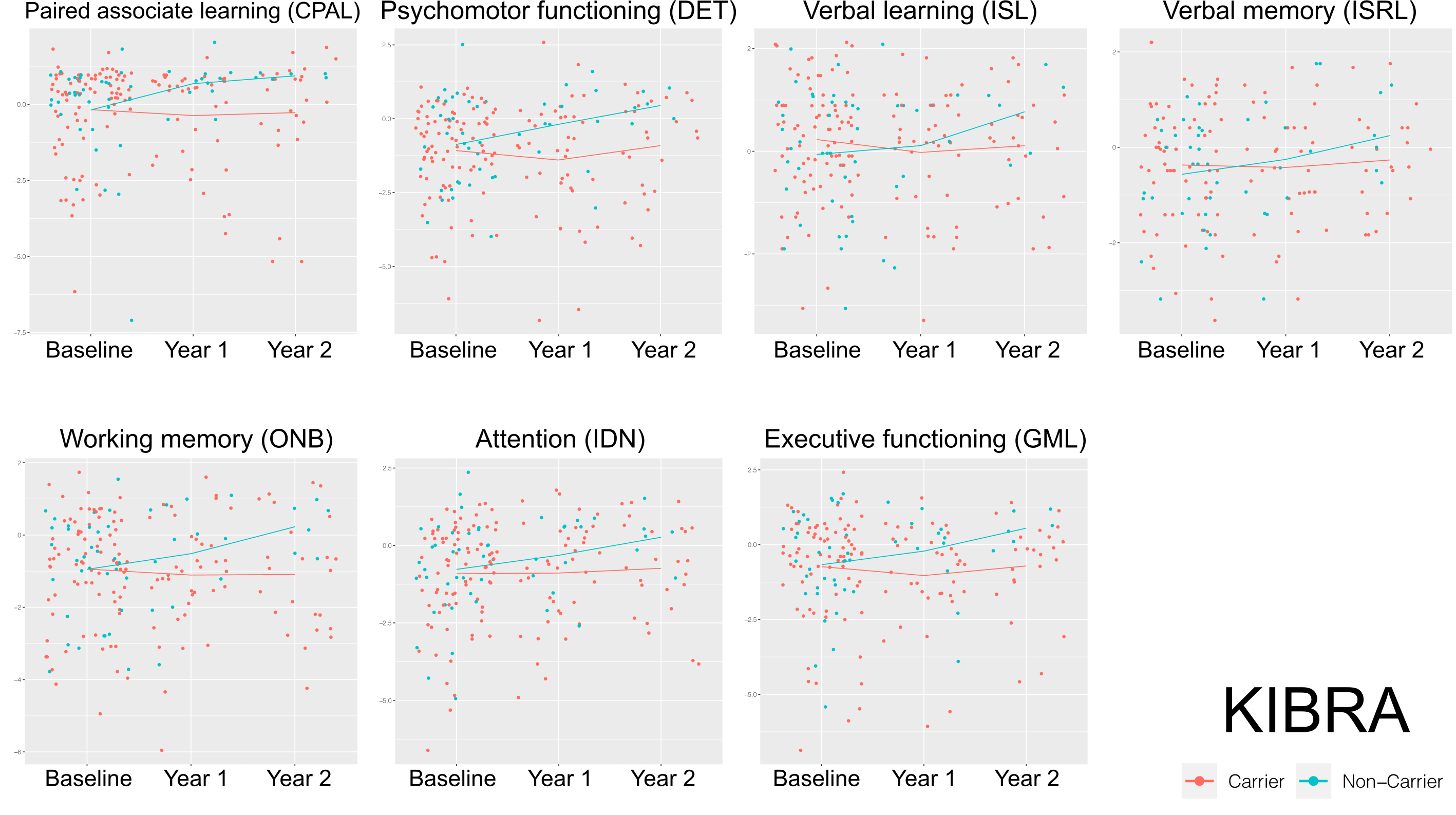

Supplement: Supplementary Figure 3 — Longitudinal impact of KIBRA carrier status across each neurocognitive domain tested at baseline, Year 1, and Year 2 of enrollment. Trajectory of KIBRA carrier versus non-carrier performance across each neurocognitive domain from initial neurocognitive testing (baseline) to timepoint 3 of neurocognitive testing (Year 2). Blue line=non-carrier, red line=carrier. [file Image_3.tiff]

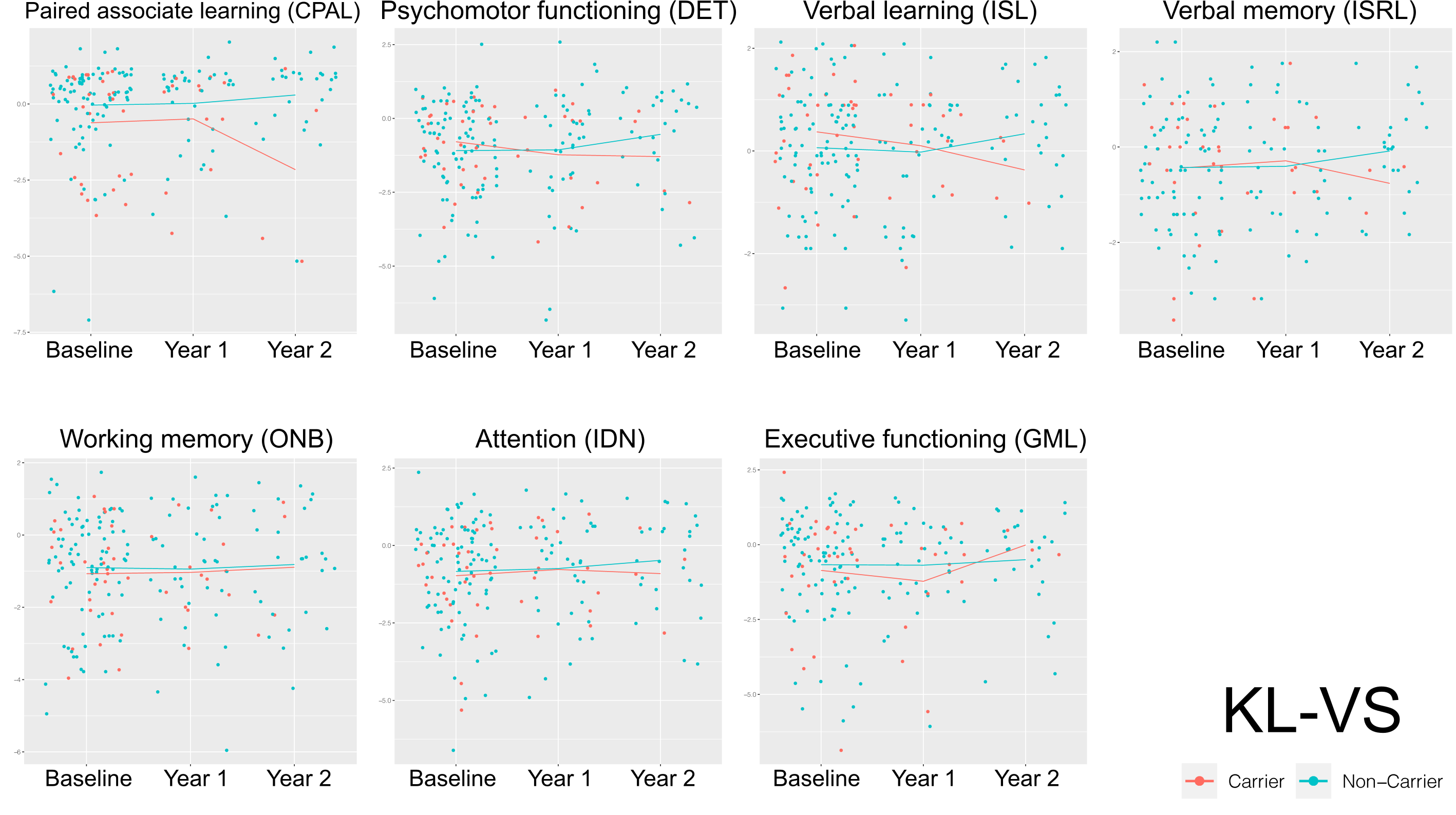

Supplement: Supplementary Figure 4 — Longitudinal impact of KL-VS carrier status across each neurocognitive domain tested at baseline, Year 1, and Year 2 of enrollment. Trajectory of KL-VS carrier versus non-carrier performance across each neurocognitive domain from initial neurocognitive testing (baseline) to timepoint 3 of neurocognitive testing (Year 2). Blue line=non-carrier, red line=carrier. [file Image_4.tiff]
