# Supplementary material for: An Integrated Analysis of Clinical, Genomic, and Imaging Features Reveals Predictors of Neurocognitive Outcomes in a Longitudinal Cohort of Pediatric Cancer Survivors, Enriched with CNS Tumors (Rad ART Pro)
Source: Front Oncol. 2022 Jun 23;12:874317. doi: 10.3389/fonc.2022.874317 (PMC9259981; doi:10.3389/fonc.2022.874317)
Supplement: Supplementary Table 1 — Description of candidate gene SNPs of interest and anticipated impact on neurocognitive outcome. Candidate gene alleles sequenced and expected effects on neurocognitive outcomes [file Table_1.docx]

**Supplemental Table 1.**

| Candidate Gene | SNP(s) | ALTERATION OF INTEREST | ADVANTAGEOUS or  DETRIMENTAL |
| --- | --- | --- | --- |
| APOE | rs429358  rs7412 | APOE ε4 heterozygote or homozygote | Detrimental |
| BDNF | rs6265 | Val66Met heterozygote or homozygote | Detrimental |
| COMT | rs4680 | Val158Met heterozygote or homozygote | Advantageous |
| KIBRA | rs17070145 | T-allele carrier | Advantageous |
| KLOTHO | rs9536314 rs95270025 | KL-VS haplotype  (Phe352Val and Cys370Ser) | Advantageous |
