# Supplementary material for: An Integrated Analysis of Clinical, Genomic, and Imaging Features Reveals Predictors of Neurocognitive Outcomes in a Longitudinal Cohort of Pediatric Cancer Survivors, Enriched with CNS Tumors (Rad ART Pro)
Source: Front Oncol. 2022 Jun 23;12:874317. doi: 10.3389/fonc.2022.874317 (PMC9259981; doi:10.3389/fonc.2022.874317)
Supplement: Supplementary Table 2 — Prevalence of candidate gene SNPs of interest across each investigated subcohort. Candidate gene allele frequencies by cohort across each row. [file Table_2.docx]

**Supplemental Table 2.**

| Candidate gene (allele of interest) | Overall Cohort | CMBs | WMLs |
| --- | --- | --- | --- |
| APOE (*ε* 4) | 17% | 15% | 11% |
| BDNF (rs6265 = A) | 36% | 32% | 46% |
| COMT (rs4680 = A) | 63% | 61% | 54% |
| KIBRA (rs17070145 = T) | 71% | 82% | 80% |
| KLOTHO (KL-VS) | 26% | 32% | 22% |
